# Sustainable waste assessment using waste biomasses in the removal of toxic textile dyes

**DOI:** 10.1038/s41598-026-53469-5

**Published:** 2026-05-28

**Authors:** Fatma Hanım Zora, Levent Gürel

**Affiliations:** 1 Department of Environmental Engineering, The Graduate School of Natural and Applied Sciences, Pamukkale University, Denizli, Türkiye; 2 Department of Environmental Engineering, Engineering Faculty, Pamukkale University, Denizli, Türkiye

**Keywords:** Toxic dye, Biosorption, Waste evaluation, Textile wastewater, Biotechnology, Chemistry, Environmental sciences, Materials science

## Abstract

Wasted tea of *Salvia officinalis* (WTSO) and the acorn cupule of *Quercus coccifera (ACQC)* were used as highly efficient biosorbents in the experiments. The aim is to convert waste materials into novel treatment materials which will be economically cheaper sources compared with conventional activated carbon. The powdered form of these materials, without any thermal or chemical pretreatment, was applied to wastewater containing Blue X GRL (BXGRL) and Red Violet 3R (RV3R) textile dyes. Various parameters affecting the separation such as pH, amount of biosorbent, contact time, dye concentration, and temperature were investigated. High removal efficiencies (up to 99%) and biosorption capacities could be readily obtained by these novel biosorbents for the studied toxic dyes. The most convenient pH and adsorbent dosage were found to be 8 and 0.1 g/100 mL, respectively. The values obtained from WTSO material with RV3R dye were fitted to Freundlich isotherm model, while the results from other material-dye sets were fitted to Langmuir model at 25 °C. The data from both ACQC and WTSO biosorbents were very well conformed to pseudo-second order reaction kinetic model with a R^2^ value of 99% when compared with pseudo first order and Elovich models. The rate limiting step was found to be chemisorption according to the results of pseudo second order and intraparticle diffusion models. In adsorption studies using high concentrations, it was observed that the dye removal efficiency increased with temperature. The highest biosorption capacities for BXGRL were obtained as 344.83 mg/g with WTSO and 222.22 mg/g for ACQC at 25 °C according to the Langmuir isotherm model. The biosorption capacities of WTSO and ACQC were 147.06 and 106.38 mg/g, respectively, for RV3R dye at 25 °C as a result of Langmuir model.

## Introduction

Climate change and the excessive use of water resources in many areas due to increasing population and developing technology have led to a significant problem: the daily depletion of clean water resources. Because wastewaters contain a wide variety of pollutants (organic substances, heavy metals, dyes, etc.), the treatment of these wastewaters is a primary priority to prevent the negative effects on aquatic organisms and human health^1^.

Textile industries consume large amounts of water during manufacturing, thus generating highly toxic wastewater that should be properly treated. The wastewaters of these industries contain various contaminants such as dyes, heavy metals, high COD and pH, salts, etc^2^. Dyes are one of the major pollutants that can render biological treatment alone insufficient for textile industry wastewater, due to their toxic characteristics and low biodegradability^3^. Many dye species are used in textile industries such as basic, acidic, direct, reactive which are soluble, and vat, sulfur, disperse, pigment which are insoluble in water^4^. There are a lot of studies carried out in the literature to separate these dyes from wastewater. Some of them are biological decolorization^5^, advanced oxidation^6^, coagulation-flocculation^7^, electro-treatment^8^, membrane processes^9^, adsorption^10^, biosorption^11^ etc.

The treatment processes except adsorption and biosorption may offer insufficient efficiency in removing dye from wastewater and are therefore less effective and also costly. Adsorption and biosorption processes are seem to be more effective in color removal from wastewaters having high removal efficiencies. These processes are also a lot of advantages such as recovery of adsorbent and contaminants after treatment process, very low effluent dye concentrations and also low costs due to environmentally friendly sorbents used^12^. These techniques were successfully used by scientists with different adsorbent materials to separate various dyes from wastewater. Some examples of sorbent materials examined in several studies are *Saccharum munja* biocomposite^13^, modified corn straw^14,15^, *Pithecellobium dulce*^16^, modified fly ash^17^, lemon peel-chitosan hydrogel^18^, natural zeolite^19^, *Helianthus annuus* L.^20^, fruit peels^21^.

The Blue X GRL (BXGRL) and Red Violet 3R (RV3R) are basic dyes used in this biosorption study. It was stated in the literature that basic dye has a structure that was more difficult to decompose than anionic dye. BXGRL can cause eye burning, difficult and rapid breathing if inhaled, a burning sensation if swallowed, wamble, methemoglobinemia, puking, too much perspiration and confusion. It was notified in the literature that this dye could be the most toxic material used in dying processes. Even so, it can be used in several dyeing processes such as cotton, silk, acrylic etc^22^. RV3R is a dye that can easily dissolve in water and has no volatility characteristics. This dye is carcinogenic and toxic for living organisms. Textile and leather industries are the known users of this dyestuff. It is harmful for humans having negative impact on derm, eye, stomach and intestine^23,24^. It has a big potential environmental risk due to these unfavorable characteristics^25^. Beside these impacts, the color appeared because of these dyes in aqueous environments is an important problem in terms of environmental esthetics.

*Quercus coccifera*, from which the acorn cupule is obtained, is a tree species found in the Mediterranean region of Türkiye. The adaptation power of this type of tree is very high. This tree is also known to spread in Denizli Province and South Aegean Region of Türkiye^26^. Furthermore, the leaves and acorns of this tree meet the energy, protein, and mineral requirements of the animals that consume them^27^. Consequently, the cupule part on the outermost shell of the acorn is a waste material. The acorn cupule of *Quercus coccifera* (ACQC) as a waste material was used in biosorption studies to see the biosorption potential. Species of *Salvia* are plants used by people in ointment, extract and tea forms for infections, cold, cold on the chest, tuberculosis, Alzheimer’s-related forgetfulness, epilepsy, stomach upsetting, etc. Also, it is used as an expectorant and carminative^28^. Sage is used in the treatment of approximately sixty different diseases worldwide. It is consumed in large quantities as an herbal tea in the Western Mediterranean and Aegean regions of Türkiye and is also exported. Türkiye is among the countries where sage is most collected^29^. In this study, wasted sage tea of *Salvia Officinalis* (WTSO) was used to assess its color removal potential.

The innovative aspect of this study can be expressed as follows: To our knowledge, there are no studies in the literature on the removal of basic dyes using adsorbents obtained from *Salvia officinalis* and cupule part of *Quercus coccifera*, which have not undergone any chemical or thermal pretreatment. Furthermore, conducting this study with actual textile dyes used in the textile industry constitutes a significant novelty. These adsorbents are waste materials, and their reintegration into the economy in terms of sustainable waste management is also an important innovation. In addition, the high removal efficiencies and retention capacities exhibited by these adsorbents despite their lack of pretreatment, and their similarly high performance at different pH values when compared with the other adsorbents used in adsorption studies, highlight the innovative aspect of this study.

In this study, the biosorption potentials of ACQC and WTSO were investigated through various tests, including the effects of initial pH, initial dye concentration, treatment time, influent temperature, and biosorbent dosage. In addition, isotherm and kinetic laboratory studies were conducted alongside morphological investigations on both biosorbents. The results were compared with those reported in the literature for the same or similar dyes and biosorbents.

## Materials and methods

Two materials which were wasted tea of *Salvia officinalis* and acorn cupule of *Quercus coccifera* were used to separate two basic textile dyes BXGRL and RV3R using biosorption technique. The information about these materials and dyestuffs, and the methodology are given below.

### Waste materials as potential biosorbents

Acorn cupules were collected from Cankurtaran location in Denizli, Turkiye. The contamination was removed from the inner and the outer parts of the cupules by washing with tap water well enough. The washing process was also applied to the cupules by putting them into a bottle and washing with tap and pure water. This process continued until the color of the pure water turned colorless. The cupules were dehumidified at 70 °C for two days and crushed to a size between 125 and 500 μm. The obtained material was dried again at 45 °C for 65 h and kept in airproof vessel. The obtained material was called ACQC anymore.

The waste sage teas were obtained from household uses of teabags of a very well-known company. Teabags were dried under the sun after the usage as a drink. The tea was separated from the bags and boiled in pure water using a magnetic stirrer with heating (Daihan Scientific MSH-20 A) until the color was removed. The suspension was filtered using a vacuum pump (LAB 312), and the material retained on the filter was dried in an oven at 70 °C for 24 h. The dried materials were milled to obtain a particle size comparable to that of acorn cupules. Dehumidification was applied again under the same conditions as before. The final material was then transferred to an airtight vessel and hereafter referred to as WTSO.

### Chemicals used in experiments

The dyes used in the experimental work have been obtained from Alfa Kimya. The molecular weights of BXGRL and RV3R are 482.57 and 368.94 g/mol, respectively. The molecular formula of BXGRL is C_20_H_26_N_4_O_6_S_2_ whereas that of RV3R is C_23_H_29_ClN_2_. The λ_max_ values of BXGRL and RV3R are 610 and 546 nm, respectively. The pH regulation agents HNO_3_ and NaOH were bought from Merck and Sigma Aldrich, respectively. These agents were used in diluted form to adjust the initial pH value of the influent. NaCl used in point of zero charge (PZC) experiments was purchased from Merck. The chemicals used for Pt-Co color analyses K_2_PtCl_6_, CoCl_2_.6H_2_O and HCl were supplied from Merck, Bostonchem and Isolab, respectively. The color analyses were conducted according to the Standard Methods (Color 2120-C). The dyes used in the experiments were of commercial grade, whereas all other chemicals were of analytical grade.

### Characterization experiments

In this work, four characterization experiments were conducted to investigate the biosorption mechanisms. These experiments are PZC, field emission scanning electron microscopy (FESEM), energy dispersive x-ray spectroscopy (EDS) and Fourier transform infrared spectroscopy (FT-IR). Dye loaded and unloaded biosorbent particles were used in these experiments to determine the functional groups and the morphology of the surfaces on the biosorbents.

Tests of PZC were made similarly to the study realized by Jaafar et al.^30^. 100 mL 0.01 M NaCl solutions were used in these tests. The pH values of these solutions were adjusted to 2–12 by using HNO_3_ and NaOH solutions. 0.125 g biosorbents were added, and the suspensions were mixed at 25 °C throughout one day in a shaker (Lab. Companion SI-300R). After the experiments, the biosorbents were separated from the suspension using a centrifuge (Hettitch Zentrifugen Universal 320). The final pH values of the solutions were analyzed and taken into a graph with initial pH values of the solutions. The PZC was determined using these graphs, which are discussed in detail in the Results and Discussion section.

Before the FESEM and EDS analyses, Quorum Q150R ES apparatus was used to coat the biosorbents with 80% Au and 20% Pd. Zeiss Supra 40VP device was utilized for FESEM and EDS analyses.

Thermo Scientific Nicolet iS50 instrument was used in FT-IR tests which were carried out using ATR (Attenuated Total Reflectance) technique. The studied wavenumber range for all biosorbents used is 400–4000 cm^− 1^ in biosorption tests.

### Modelling

Isotherm and kinetic modelling of the experimental data carried out using several isotherm and kinetic models. The models and the equations used in this part of the study are presented in Table 1.

### Experimental progression

The basic dyes BXGRL and RV3R were used to prepare model textile wastewater using known amounts in pure water. The dye concentrations were nearly 50 mg/L in all studies except initial dye concentration test (variable in the range of 25–300 mg/L). In the pH variation studies, 0.5 g of biosorbents were added to 100 mL of dye solutions with an initial concentration of approximately 50 mg/L, adjusted to the desired pH. The pH range studied is between 2 and 10. The amount of biosorbent was varied in biosorbent dosage studies (approximately in the range of 0.01–0.75 g/100 mL). 0.1 g biosorbent was used for 100 mL of wastewater in contact time and initial dye concentration tests. The experiments were conducted for 24 h in the pH and biosorbent dosage studies, 350 min in the contact time experiments, and 18 h in the initial dye concentration studies. The initial dye concentration experiments were conducted at different temperatures (25, 40, and 55 °C), whereas all other experiments were performed at 25 °C. A brief explanation for the biosorption studies is given below.

**Table 1:** Equations of biosorption kinetic and isotherm models.

Models	Equation	Parameters	Reference
Isotherm models			
Langmuir	$\:\frac{{C}_{e}}{{q}_{e}}=\frac{{C}_{e}}{{q}_{max}}+\frac{1}{{q}_{max}.{K}_{L}}$ $\:{R}_{L}=\frac{1}{1+{K}_{L}.{C}_{0}}$	q_max_: maximum uptake capacity K_L_: constant of equilibrium R_L_: separation factor	^47^
Freundlich	$\:{log}{q}_{e}={log}{K}_{F}+\frac{1}{n}{log}{C}_{e}$	K_F_: constant of Freundlich n: coefficient of Freundlich	^18^
Temkin	$\:{q}_{e}=B{ln}{K}_{T}+B{ln}{C}_{e}$ $\:B=\frac{RT}{{b}_{tem}}$	K_T_: constant of equilibrium b_tem_: constant of Temkin isotherm	^49^
Dubinin-Radushkevich	$\:{ln}{q}_{e}={ln}{q}_{max}-{K}_{D\&R}.{\epsilon\:}^{2}$ $\:\epsilon\:=RT{ln}\left(1+\frac{1}{{C}_{e}}\right)$ $\:E=\frac{1}{\sqrt{2{K}_{D\&R}}}$	$\:\epsilon\:$: Polanyi sorption potential K_D&R_: constant of isotherm E: mean free energy	^24^
Kinetic models			
Pseudo-first-order	$\:{log}\left({q}_{e}-{q}_{t}\right)={log}{q}_{e}-{k}_{pf1}.t$	k_pf1_: rate constant	^58^
Pseudo-second-order	$\:\frac{t}{{q}_{t}}=\frac{1}{{k}_{pf2}.{q}_{e}^{2}}+\frac{1}{{q}_{e}}.t$	k_pf2_: rate constant	^45^
Elovich	$\:{q}_{t}=\frac{1}{\beta\:}{ln}\alpha\:\beta\:+\frac{1}{\beta\:}{ln}t$	$\:\beta\:$: constant of desorption $\:\alpha\:$: rate of initial adsorption	^32^
Weber Morris	$\:{q}_{t}={k}_{wm}.{t}^{1/2}+c$	k_wm_: rate constant of intraparticle diffusion c: intercept of y-axis	^55^

A certain amount of biosorbent was added to the pH adjusted 100 mL dye containing wastewater. The suspension which was formed was put in a shaker and the shaker speed was adjusted to 150 rpm, and it was operated along necessary contact time until the biosorption equilibrium obtained. After the required contact time, the suspension was subjected to centrifugation process at a speed of 5000 rpm for 5 min to separate biosorbent from the water. The solution (free from biosorbent) pH and absorbance value were measured using a pH meter (WTW Multi 3420) and an UV spectrophotometer (Hach Lange DR500), respectively. And the concentration of the dye solution was determined using the calibration curve.

## Results and discussion

### Characterization tests

In this part, four characterization studies were conducted to investigate the biosorbent surface properties and biosorption mechanisms in detail. These studies have been explained in further sections below.

#### PZC analysis

**Fig. 1:**
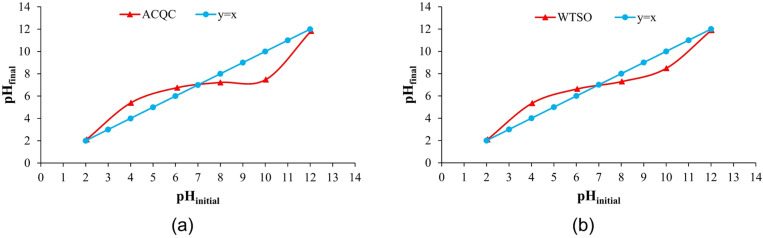
PZC characterization test results for ACQC (**a**), and WTSO (**b**).

The surface charge of the biosorbent at different pH values was observed by PZC tests. The initial pH values of the salt solution measured before adsorption and the final pH values measured after a sufficient contact period in the shaker were plotted graphically. The initial pH (pH_initial_) values were plotted against final pH (pH_final_) values in the graph. The graphs of PZC study for ACQC and WTSO are exhibited in Fig. 1.

A similar trend was observed for both biosorbents studied. According to the results of the studies for ACQC and WTSO, it was clear that the PZC value for both biosorbents was approximately 7.0. Above this value, it was seen that the surfaces of the biosorbents were charged negatively while they were positively charged below pH 7. This can be explained by the fact that functional groups, such as carboxyl and hydroxyl groups, which play an important role in adsorption, lose their protons. Electrostatic interactions are very important in this case. The details of the effects of electrostatic forces and the other interactions can be found in the literature^1,31^. Negatively charged biosorbent surface can capture dye molecules which have cationic behavior. So, the best biosorption performance was expected above 7 pH for cationic dyes used in the study. It is generally considered that pH values below 7 are more suitable for the removal of anionic dyes. Examples illustrating the effects of lower and higher pH values on the removal of anionic and cationic dyes can be found in the literature^32,33^.

#### SEM and EDS analyses

**Fig. 2:**
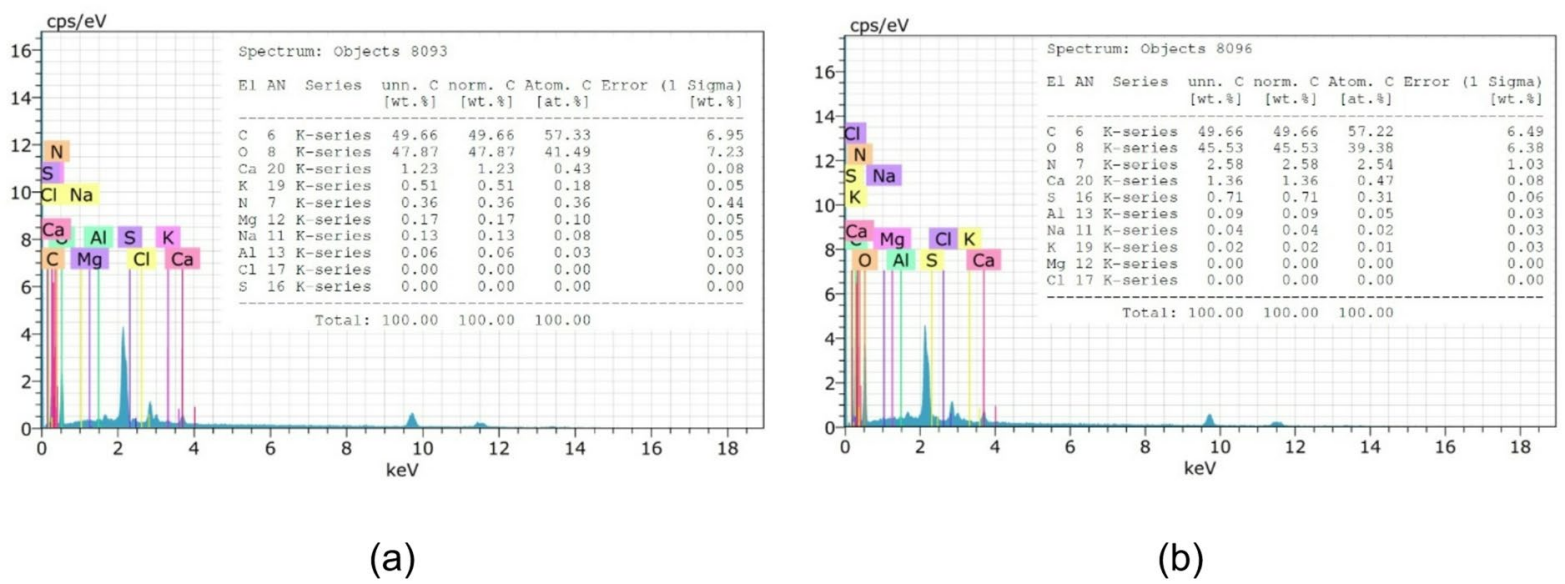
EDS analyses results for raw ACQC (**a**) and raw WTSO (**b**).

**Fig. 3:**
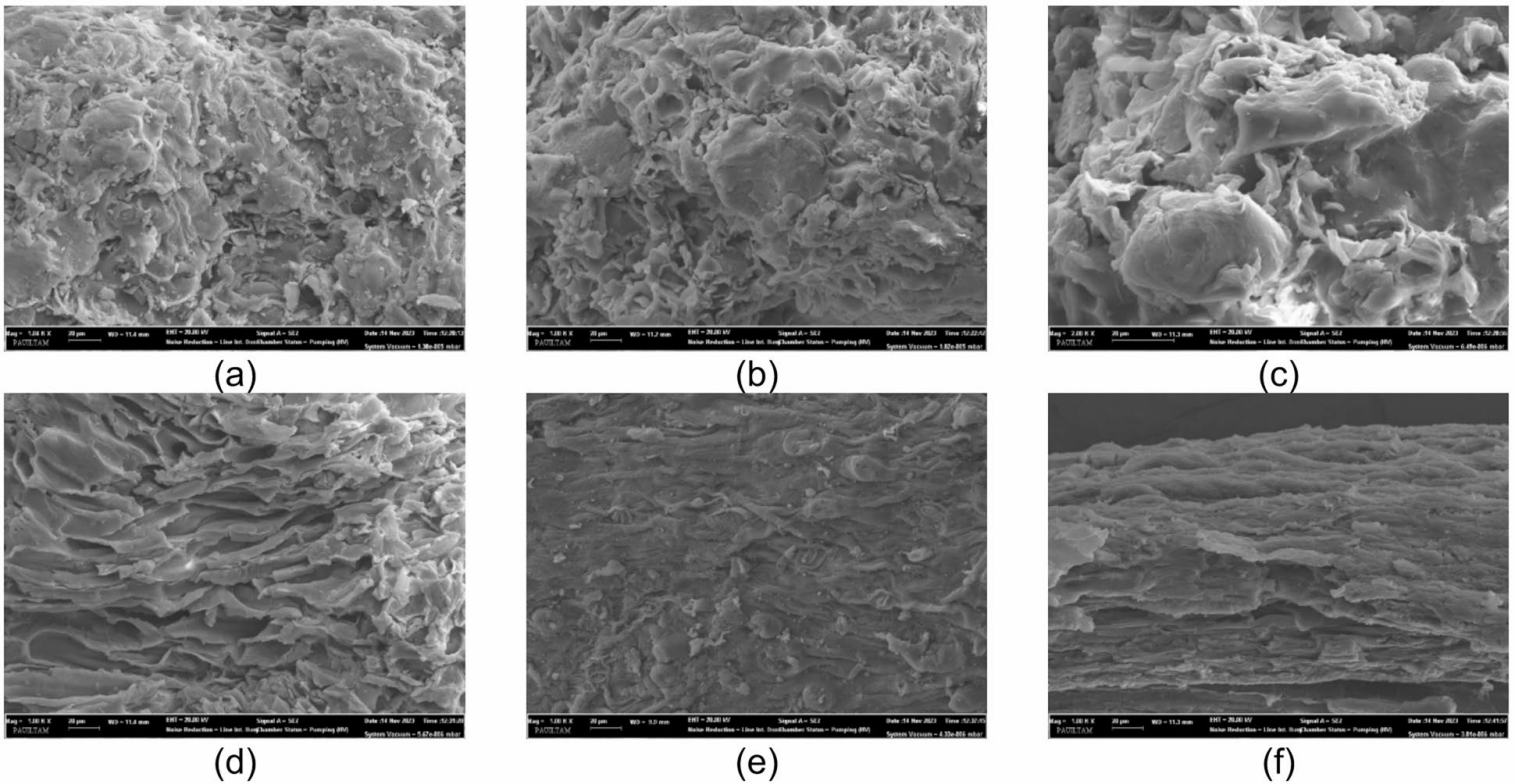
Scanning electron microscopy images of raw ACQC (**a**), ACQC with BXGRL (**b**), ACQC with RV3R (**c**), raw WTSO (**d**), WTSO with BXGRL (**e**), and WTSO with RV3R (**f**).

Surface properties of biosorbents are very important in biosorption studies due to morphological structure and functional groups found on the surface. To analyze the characteristics of the biosorbent surface, SEM and EDS analyses were conducted. The results of the EDS analyses are given in Fig. 2. The photos taken using FESEM device are also presented in Fig. 3.

According to the results obtained from EDS, it was observed that both ACQC and WTSO contain various elements such as C, O, Ca, N, K, Na in common. Carbon and oxygen constitute a large part of the biosorbent materials. WTSO has differentiated from the ACQC with higher amounts of nitrogen and sulfur on its surface. The amount of calcium, as well as the amount of carbon and oxygen, is at the same level in both biosorbents. These elements may be decisive for the functional groups which are effective in biosorption phenomena. In SEM photos of Fig. 3, it can be clearly seen that a notched, irregular and protruding surface structures are present on both biosorbents. A heterogeneous surface was obviously observed from micrographs for all biomasses. While the surfaces of the biosorbents were rugged and rough before the biosorption process, the dye dispersed on the surface and caused several changes in comparison with the initial photo after the biosorption process. Similar cases were reported in the literature which the natural biosorbents used in biosorption studies^21^. Also the porous structure of both biosorbents can be readily observed from SEM images. The analyses revealed that the voids and irregular surfaces observed on the biosorbent surface play an effective role in the physical adsorption of textile dyes by improving the existing surface area and creating regions exhibiting additional active properties^1^. It can be concluded by the FESEM studies that the biosorption was realized successfully by adsorbing toxic dyes on the surface of the biosorbents.

#### FTIR analysis

**Fig. 4:**
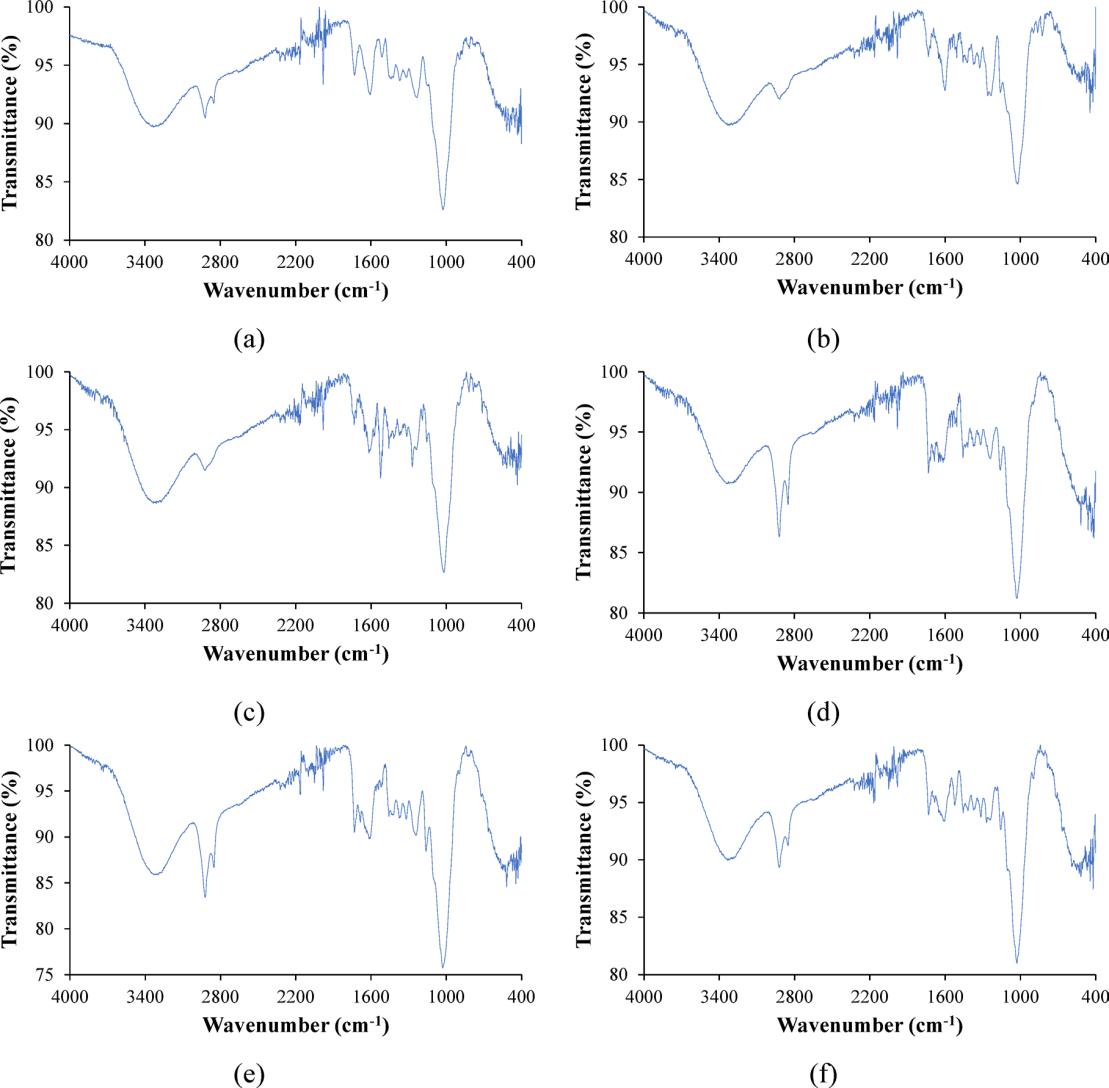
The results of FTIR analyses for raw ACQC (**a**), ACQC with BXGRL (**b**), ACQC with RV3R (**c**), raw WTSO (**d**), WTSO with BXGRL (**e**), and WTSO with RV3R (**f**).

These analyses were conducted with unused and used biosorbents to determine the functional groups on the surfaces and to investigate the changes on biosorbents after separation of dye by biosorption. The results achieved by FTIR analyses are shown in Fig. 4 for unloaded and loaded biosorbents with toxic dyes used in studies.

After the experiments there are many absorption peaks in FTIR results which show the complex structure of the biosorbents studied. The important peaks obtained from the device are 3330, 2919, 2851 1730, 1606, 1370, 1320, 1233, 1027 cm^− 1^ for ACQC in raw form, while peaks of 3332, 2922, 2851, 1733, 1457, 1374, 1318, 1238, 1160 and 1027 cm^− 1^ for WTSO in raw form. It is seen that the peaks obtained for two unloaded biosorbents are similar. The peaks at 3330 and 3332 cm^− 1^ are interested in O-H groups for both biosorbents^34^. C-H stretching comes to mind with the peaks observed at 2851, 2919 and 2922 cm^− 1^. It is known that this bond can usually take part in methyl groups of the material^35^. Bands at 1730 and 1733 cm^− 1^ expresses C = O stretching^32^. According to last mentioned peaks, carboxyl, aldehydes, ketones or esters which are simple carbonyl compounds can be found on the surface of the materials^36^. Between 1300 and 1420 and 1550–1610 cm^− 1^, carboxylate functional groups were identified in the literature which conformed to the data obtained for two biosorbents^37^. The bands of 1027, 1233 and 1238 cm^− 1^ can be attributed to the C-O bonds which correspond to the groups of alcohol, phenol and ether may be present on the surfaces of the biosorbents^35,38^. After the dye separation process, the peaks for the biosorbents loaded with dyes moved higher or lower wavenumbers as it can be seen from Fig. 4-b, c, e, f showing the success of functional groups found on the surface of biosorbents. Because of the results mentioned above, it can be said that various functional groups such as carboxylate, hydroxyl, carbonyl etc. which are thought to be effective at the biosorption process played a major role on the separation of toxic dyes from dye containing wastewater. Carbonyl groups on the surface of the adsorbent positively effect π–π stacking interactions. The adsorption performance of the biosorbent increases. Also high removal efficiencies can be achieved even with increased initial dye concentrations. Several interaction mechanisms, such as π–π interactions and electrostatic forces, are believed to play a significant role in the adsorption process^1,31^.

### The effects of operating parameters

There are many parameters that can affect all biosorption process such as initial pH value of wastewater, the amount of biosorbent used in solution to be treated, the contact time of dye solution with biosorbent material, initial toxic dye concentration, temperature of the treated solution, etc. To identify the entire biosorption process, the parameters mentioned above were studied in detail and presented in the following sections.

#### The initial pH of wastewater

**Fig. 5:**
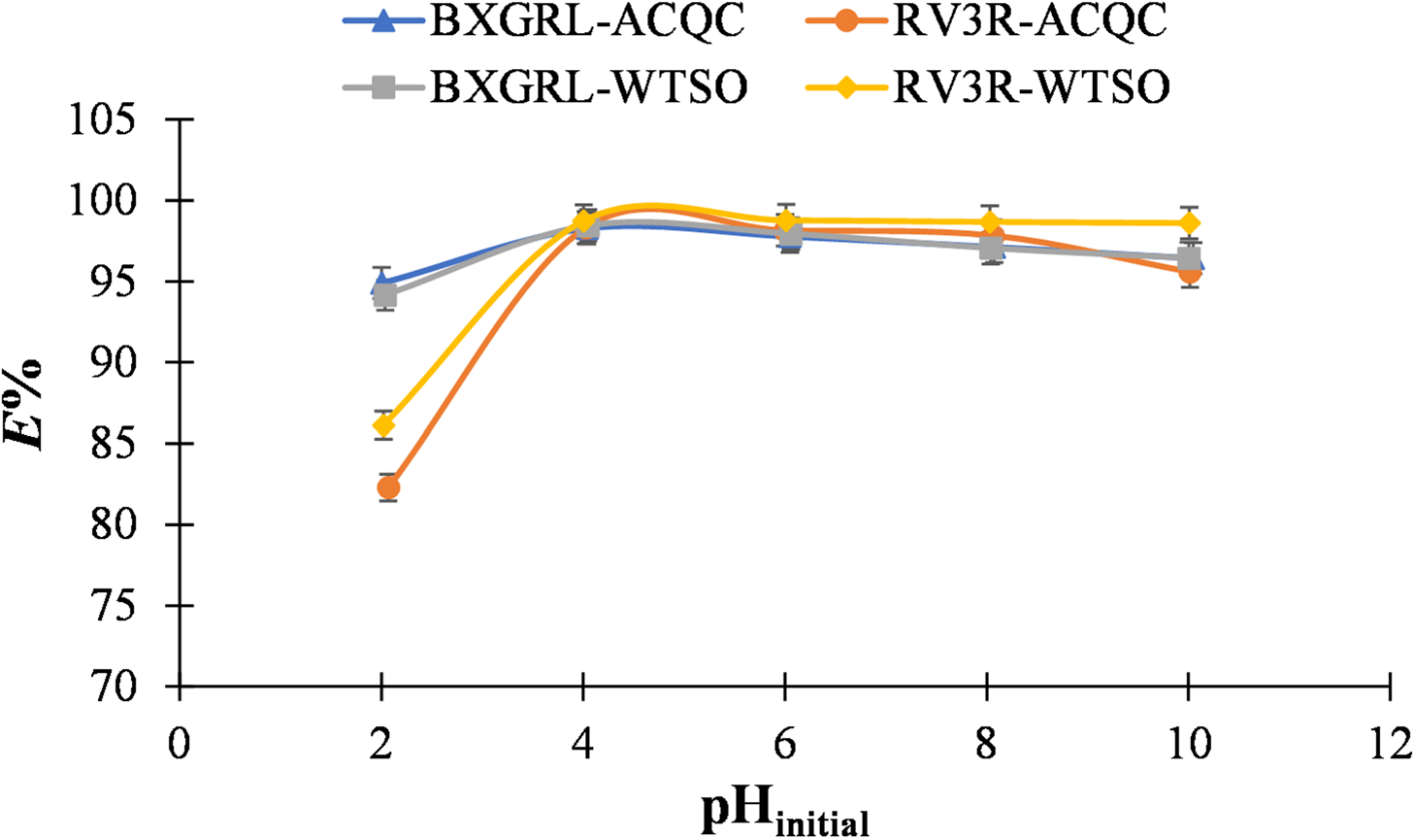
The pH effect on separation efficiency of ACQC and WTSO.

To investigate the effect of initial pH value of toxic dye solutions, five different pH values were used in pH studies such as 2, 4, 6, 8 and 10. The solutions were adjusted to the planned pH by using diluted HNO_3_ and NaOH solutions before biosorption tests. The importance and the effective pH interval of the biosorbents on toxic dye removal were evaluated. The outcomes of the pH study are presented in Fig. 5 in the scope of the separation efficiency of biosorbents.

The test results showed that all biosorbents studied were very effective in pH range of 4–10 which was very important for wastewaters of textile industries. This wide pH range enables the biosorbent to be applied to wastewater containing different types of dyes. The highest biosorption efficiency was found to be 98.77% at pH 6 with WTSO-RV3R. The lowest removal efficiencies were observed at pH 2 for all biosorbents and dyes studied. It can be said that the separation performance between 4 and 10 pH is very high and very close to each other. The highest concentrations remained in the solution after biosorption was detected as 2.72, 4.56, 3.03 and 2.44 mg/L at pH 2 for the sets BXGRL-ACQC, RV3R-ACQC, BXGRL-WTSO and RV3R-WTSO, respectively. At lower pH values, a competitive biosorption could take place in separation process due to high amount of hydronium ions mixed into synthetic wastewater during pH adjustment of the initial dye solution. As a result of this competition, the cationic dye separation efficiency decreases dramatically at this pH values due to the blocking of several functional groups effective on dye sorption^39^. The PZC value was detected as 7 for two biosorbents. The surface of the biosorbent was positively charged under pH 7 according to the PZC studies which supported the lower removal efficiencies around pH 2. It is expected that the surface of the biosorbents can contain several functional groups such as hydroxyl and carboxylates which could be effective on biosorption of cationic toxic dyes above zero charge point^40^. Because of this negatively charged biosorbent surface and the commonly basic characteristics of textile wastewater and the separation efficiencies found very close to each other, the most favorable pH was chosen as 8, and the other studies were conducted at this value. At some studies in the literature similar pH values such as 7 and 8 found to be optimum to remove basic dyes from the dye containing solutions^22,39,41^.

#### The amount of biosorbents

**Fig. 6:**
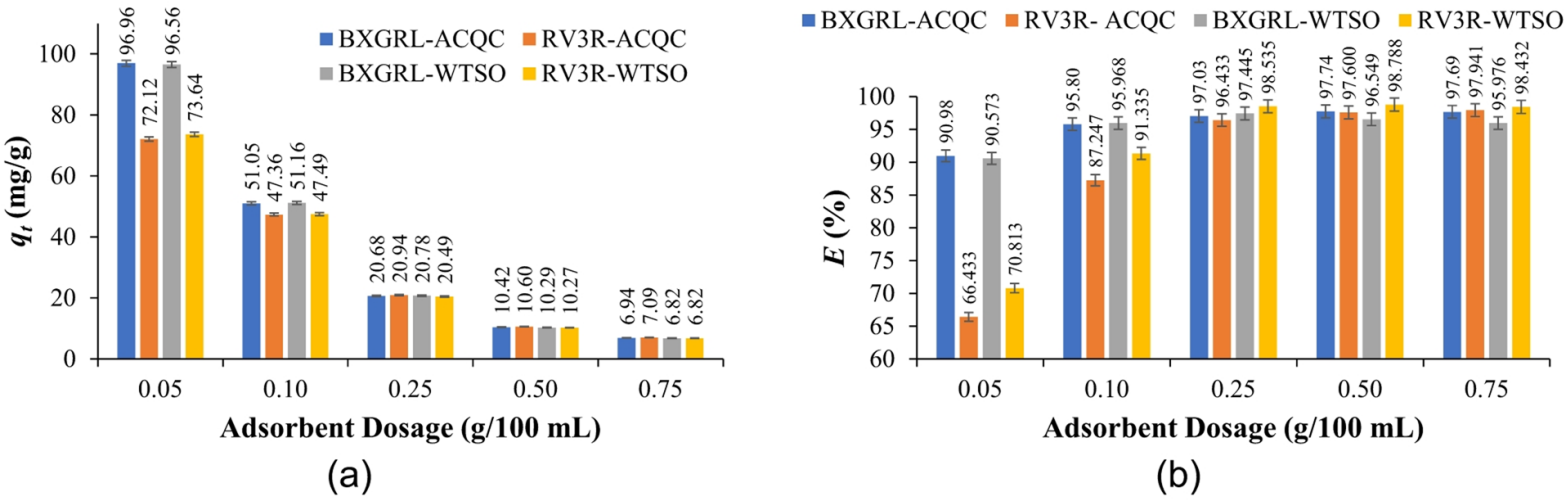
The influence of the amount of biosorbent on sorption capacity (**a**) and biosorption efficiency (**b**).

In the studies conducted to remove color from real wastewater, it is very important to adjust the correct biosorbent dosage for efficient color removal and dye separation. Data of isotherm studies must be obtained to adjust the optimum dosage for a given initial concentration of dye solution. Different amounts of biosorbents within the range of 0.05–0.75 g per 100 mL were used to answer this question. The biosorbent separation efficiencies and results of sorption amounts are given in Fig. 6.

The data obtained from the studies showed that the highest sorption capacity and the lowest removal efficiency for both toxic dyes were recorded at the lowest biosorbent dosage. The biosorption capacity was decreased while the separation efficiency increased at higher dosages of the material per 100 mL of dye solution. This phenomenon was observed in various studies carried out in literature^42,43^. When two biosorbents were compared to each other for dosages under 0.25 g/100 mL, it can be obviously comprehended that the separation efficiencies were very close to one another. However, BXGRL was much more efficiently removed against RV3R at mentioned dosages. At lower biosorbent dosages, most of the active sites on the biosorbent surface become occupied by the end of the sorption process, resulting in a limited number of free sites. So, the biosorbent surface is efficiently loaded with the target solute, and this maximizes the amount of dye adsorbed per gram of biosorbent materials. Increasing the biosorbent dosage leads to a higher number of unoccupied biosorption sites at the end of the reaction period, as the dye concentration is kept constant while the amount of biosorbent is relatively high for this concentration level. As a result, the uptake capacity decreases with increasing biosorbent dosage, and the biosorbent material cannot be loaded efficiently. Conversely to the trend of sorption capacity, separation efficiency rises with increasing biosorbent dosage because the constant dye concentration can be fully adsorbed on the surface of biosorbent which is very high in quantity than necessary. The results obtained and the comments made in this study are compatible with the studies in the literature^44,45^.

#### Contact time of adsorbate with biosorbent

**Fig. 7:**
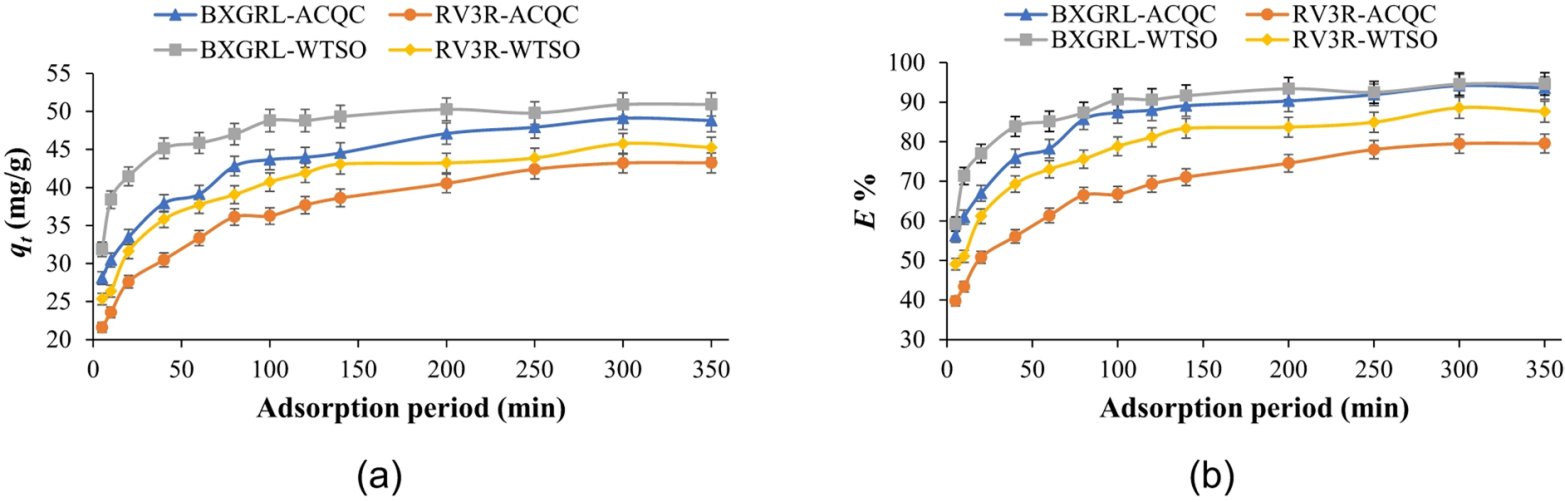
Sorption capacity (**a**) and the biosorption efficiency (**b**) variation with the time of biosorption process.

The rate of a separation process is very important for the use in a big scale continuous treatment system and for the design of the continuous separation process. Various tests were carried out to investigate the speed of the biosorption by ecofriendly biosorbents. The data found from the time tests are placed in Fig. 7.

The rate of the sorption process is very high at the beginning of the treatment process. After fifty minutes of the biosorption period, the rate of the process decreased, and the equilibrium was nearly reached after 150 min of treatment. The equilibrium was obtained at 200 min in the set of BXGRL-WTSO while at 300 min in other sets. The data reveal that the general trend for all sets is similar. The highest separation efficiencies were obtained for the removal of BXGRL dye with ACQC and WTSO as 94.16% and 94.58%, respectively at 300 min of process. The rate of the process is very high at the beginning of the biosorption period due to the large quantity of free biosorbent surface found on the biomass. In the later periods of treatment, these free biosorption sites began to be saturated, and the biosorption rate decreased in this way. Similar results are also found in the other studies regarding the biosorption of impurities^46,47^. In a study, the equilibrium time was found to be 210 min for modified clinoptilolite removing basic blue 41^40^. In another work conducted to remove the basic blue 41 using eucalyptus bark and composites, equilibrium was reached at 90 min of biosorption process^48^. Olive leaf extract was used to obtain Fe_3_O_4_ nanoparticles in a work to decolorize the solution containing basic violet 16, and the equilibrium was recorded at 210 min^49^.

#### Initial concentration of toxic dyes and temperature of aqueous media

**Fig. 8:**
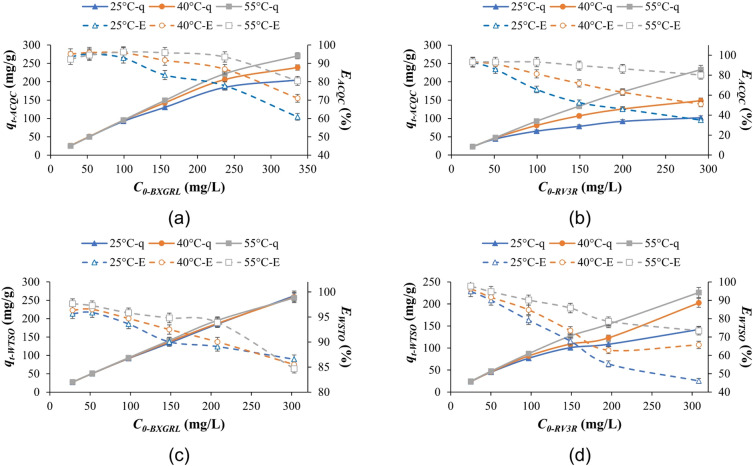
The influence of initial dyestuff concentration on sorption capacity and separation efficiency for ACQC with BXGRL (**a**), ACQC with RV3R (**b**), WTSO with BXGRL (**c**), and WTSO with RV3R (**d**).

The dyes used in textile sector can be mixed with the wastewater of the plant in different quantities. To assess the performance of the constant biomass dosage for lower and higher initial dye concentrations, analyses were conducted with dye concentrations in the range of 26–310 mg/L approximately. Also, different temperatures such as 25, 40 and 55 °C were also evaluated at the concentrations of dye mentioned above. The results of these temperature and dye concentration studies are demonstrated in Fig. 8.

It can be clearly seen from Fig. 8 that the separation efficiencies for all dyes and biosorbents are very high at lowest initial dye concentrations. In contrast to efficiencies, sorption capacities of the biosorbents are at the lowest value in all tests conducted in this section. The lowest separation efficiency was obtained at an initial RV3R (for ACQC) concentration of 291 mg/L as 35% at 25 °C. The highest separation efficiency was also found to be 97.7% at approximately 25 mg/L in the set of WTSO-RV3R conducted at 55 °C. The best biosorption capacity was achieved to be 270.58 mg/g with ACQC biosorbent treating 336 mg/L BXGRL at 55 °C. The worst capacity was also observed with ACQC as 23.04 mg/g at an initial RV3R concentration of 25 mg/L at 40 °C. As can be seen from results of separation efficiency and uptake capacity studies, with the increase in the initial dye concentration, the biosorption capacity increases and the separation efficiency decreases. The increase in biosorption capacity with increasing dyestuff concentration can be explained by the effective coating of the surface of the biosorbent with high amounts of dye molecules at a fixed biosorbent dosage.

It can also be concluded that mass transfer occurs more efficiently because of higher dye concentration. The decrease in separation efficiency can be explained by the fact that there is not enough surface left on the biosorbent particles at fixed dosage to adsorb dye molecules at high concentrations. In this case, a significant portion of the dye molecules remain untreated in the water, resulting in a decrease in separation efficiency. Similar results were observed in various studies in the literature^40,50^. It was observed that the removal efficiency of BXGRL dye with both biosorbents remained higher compared to RV3R dye with increasing initial dye concentration. The best removal efficiencies against increasing dye concentration were obtained in the set where BXGRL removal was performed with WTSO biosorbent. When the biosorption temperatures were evaluated, it was observed that there was not much variability in the removal efficiencies at low dye concentrations, and that the difference in the removal efficiencies depending on the temperature increased when the dye concentration increased. This situation was also seen in the biosorption capacities in a similar way, except for the WTSO-BXGRL set. The effect of temperature on the biosorption capacities was limited in the WTSO-BXGRL set.

### Modelling tests

Data of contact time and initial dye concentration studies were modelled using different kinetic and isotherm models to analyze the mechanism of the dye biosorption process.

#### Isotherms

Isotherm studies are important to have information about the biosorbent dosage to be used at different wastewater influent concentrations, to interpret the biosorption mechanism and to evaluate the compatibility of the biosorbent with the contaminant. The parameters obtained from experiments and calculated from several models are given in Table 2.

**Table 2:** Isotherm models for the biosorption of dyes with ACQC and WTSO biosorbents.

Models and parameters		Dyes&Biosorbents											
		BXGRL-ACQC			BXGRL-WTSO			RV3R-ACQC			RV3R-WTSO		
Temperature (°C)		25	40	55	25	40	55	25	40	55	25	40	55
Langmuir	*q*_*max*_ (mg/g)	222.22	263.16	333.33	344.83	322.58	294.12	106.38	158.73	303.03	147.06	196.08	243.90
	*K*_*L*_ (L/mg)	0.082	0.102	0.072	0.060	0.081	0.135	0.067	0.066	0.053	0.063	0.053	0.071
	*R* ^*2*^	0.9957	0.9979	0.9249	0.9274	0.9819	0.9965	0.9884	0.9918	0.9919	0.9764	0.8602	0.9429
Freundlich	*n*	2.23	2.03	1.61	1.63	1.72	1.85	3.24	2.43	1.56	2.79	2.43	2.25
	*K*_*F*_ (mg/g)(L/mg)^1/n^	28.52	33.40	29.30	27.38	31.92	40.94	21.37	21.69	20.84	23.79	25.36	31.40
	*R* ^*2*^	0.9065	0.8894	0.7635	0.9860	0.9803	0.9592	0.9837	0.9661	0.9565	0.9843	0.9768	0.9976
Temkin	*B*	41.63	51.274	73.204	61.864	59.702	56.451	16.536	28.309	59.194	23.292	31.557	38.106
	*K*_*T*_	1.171	1.331	0.869	0.966	1.206	1.877	2.036	1.135	0.726	1.657	1.389	1.837
	*R* ^*2*^	0.9855	0.9884	0.9381	0.9251	0.9650	0.9711	0.9857	0.9944	0.9857	0.9723	0.8316	0.9031
D-R	*q*_*max*_ (mg/g)	151.53	170.51	230.60	155.09	157.48	154.36	75.42	103.41	144.71	92.16	104.67	119.06
	*K*_*DR*_ (mol^2^/kj^2^)	1.5229	0.9748	2.091	0.8773	0.6165	0.3006	0.9436	1.2031	1.2311	0.6856	0.4032	0.2150
	*E* (kJ/mol)	0.573	0.716	0.489	0.755	0.901	1.290	0.728	0.645	0.637	0.854	1.114	1.525
	*R* ^*2*^	0.9125	0.9032	0.9793	0.8197	0.8314	0.7913	0.7768	0.8176	0.8267	0.7356	0.6537	0.6606

As a result of the calculations carried out with the data obtained from the experimental results, it was understood that all other biosorbent and dye sets used in the removal of RV3R and BXGRL dyes, except WTSO with RV3R, were compatible with the Langmuir isotherm. It was observed that the WTSO-RV3R removal process gave more compatible results with the Freundlich model among all the isotherm models studied. At the same time, it was observed that the Freundlich isotherm gave better results when the *R*^*2*^ values were considered at 25 °C in the BXGRL-WTSO set. In the BXGRL dye tests performed with both ACQC and WTSO biosorbents, it can be stated that the surfaces have homogeneous properties, the biosorption can be expressed as monolayer according to the Langmuir isotherm model. In the set of RV3R-ACQC a similar trend was observed. However, according to the Freundlich isotherm model in the removal of RV3R with WTSO, it is thought that the biosorbent exhibits heterogeneous properties against the adsorbate and the biosorption is not limited to a single layer on the surface of the material. It is observed that there is no significant agreement between the Temkin and Dubinin-Radushkevich isotherm models at 25 °C and almost all other temperatures. Considering the Langmuir isotherm data, it was determined that the maximum biosorption capacity was obtained in the biosorption of BXGRL dye with WTSO at 25 °C and this value was 344.83 mg/g. It is understood from Table 2 that the q_max_ value decreases as the temperature increases in the BXGRL-WTSO set. While the experimental biosorption capacity value increases with temperature at the initial BXGRL concentrations of 28, 53, 98, 148 and 208 mg/L used in the study, the experimental biosorption capacity value decreases with temperature increase only at the initial BXGRL concentration of 303 mg/L. It is thought that one of the reasons for the decrease in *q*_*max*_ values obtained from the model due to temperature increase is this decrease in the experimental biosorption capacity occurring at the highest initial concentration. The separation factor (*R*_*L*_) was also calculated using the data obtained from laboratory studies and the Langmuir constant, *b*. In the calculations performed, separation factors varied between 0.0239 and 0.4323 in all sets studied showing the favorable biosorption process (0 < *R*_*L*_<1). The Freundlich parameter, *n* gives information about the favorability of the biosorption and heterogeneity of the biosorbent surface. According to the literature, value of 1/*n* between 0 and 1 shows the favorable biosorption process of the biosorbent^51,52^. In this context, the 1/n values calculated in the WTSO-RV3R set, where the Freundlich isotherm is valid, were found to be 0.358, 0.412 and 0.444 for 25, 40 and 55 °C, respectively. When Table 2 is examined, it is clearly understood that the 1/*n* values obtained from the other sets also remain in the range of 0 and 1.

#### Kinetics

The rate of biosorption process is very curious in the design of separation processes. It is possible to get information about the mechanism of biosorption process by kinetic studies. Physisorption, chemisorption and diffusion properties of the biosorption process can easily be determined with these kinetic model computations. The calculated parameters of the studied kinetic models are presented in Table 3.

When the results obtained from the model calculations are examined, it can be clearly seen that the *R*^*2*^ values belonging to the pseudo-second-order kinetic model are higher than all other studied models. The experimental sorption capacities are also compatible with the uptake capacities attained from pseudo-second-order model showing the most appropriate kinetic model for the biosorption of two toxic basic dyes. Although the *R*^*2*^ values of the pseudo-first-order kinetic model are high, the biosorption capacities found with this model are considerably lower than the capacities obtained experimentally.

**Table 3:** Kinetic models for the biosorption of BXGRL and RV3R dyes by ACQC and WTSO.

Models and parameters		Dyes & Biosorbents			
		BXGRL-ACQC	BXGRL-WTSO	RV3R-ACQC	RV3R-WTSO
*q*_*e*_ (mg/g) _(experimental)_		49.09	50.27	43.21	45.75
Pseudo first order	*q*_*e*_ (mg/g)	19.34	14.84	21.57	16.25
	*k*_*pfo*_(1/min)	0.011	0.020	0.012	0.010
	*R* ^*2*^	0.9850	0.9645	0.9733	0.9167
Pseudo second order	*q*_*e*_ (mg/g)	50.00	51.02	44.44	46.30
	*k*_*pso*_ (g/mg.min)	0.0017	0.0041	0.0015	0.0020
	*R* ^*2*^	0.9978	0.9995	0.9962	0.9984
Elovich	*β* (g/mg)	0.1873	0.2111	0.1821	0.1906
	*α* (mg/g.min)	169.43	1238.04	44.06	111.36
	*R* ^*2*^	0.9901	0.9662	0.9908	0.9847
Weber-Morris	*k*_*WM*_ (mg/g.min^0.5^)	1.37	1.37	1.42	1.32
	*c* (mg/g)	27.77	33.75	20.91	25.46
	*R* ^*2*^	0.9330	0.8436	0.9491	0.8885

This shows that the pseudo-first-order kinetic model is insufficient to describe this biosorption process. Both Elovich and Weber-Morris models have lower *R*^*2*^ values than pseudo-second-order model. At the same time, for the rate-limiting step of biosorption to be intraparticle diffusion, the Weber-Morris model (*c*) coefficient must be zero, that is, the resulting model curve must pass through the origin. When the c values in Table 3 are examined, it can be clearly seen that such a situation is not the case. In line with all the above-mentioned issues, it was concluded that the model that best fitted the test results was the pseudo-second-order kinetic model, and the probable biosorption mechanism is chemisorption. Although intraparticle diffusion alone is not the rate-limiting step, model results indicate that a portion of the dye is adsorbed within the pores, which can be considered a significant factor in increasing adsorption efficiency. Indeed, this was visually observed through SEM analysis^31,53^. Accordingly, electrons may have been shared or exchanged between toxic dyes and biosorbents. In this case, it can be stated that covalent forces and ion exchange may be effective in biosorption of toxic basic dyes^54^. Studies with similar dyes and biosorbents found in the literature^39,48,55^ showed that the most appropriate model in biosorption is pseudo-second-order model as in this study.

### Comparison of biosorbents and dyes

In this section, the toxic dye biosorption capacities of two different biosorbents were compared with each other as well as with results reported in the literature. The results of this work and the other literature studies are exhibited in Table 4.

**Table 4:** A literature survey and comparison of different biosorbents on dye removal.

Adsorbent& pollutant	C_0_ (mg/L)	m (g/L)	T (°C)	q_max_ (mg/g)	Reference
Seeds of *Salvia sclarea*& Basic Violet 16	10–300	10	RT	19.80	^23^
Natural clinoptilolite& Basic Blue 41	20–200	1	50	149.25	^40^
Modified clinoptilolite& Basic Blue 41	20–200	1	50	370.37	^40^
MG-*Eucalyptus bark*	200	0.05	27	186.70	^48^
Sunflower seed shells& Red X-5GN	27–210	10	25	27.40	^20^
Sunflower seed shells& Reactive Blue 221	43–382	4	25	30.58	^20^
Fe_3_O_4_ nanoparticles & Basic Violet 16	10–50	0.8	45	117.64	^49^
Tea waste& Acid Blue 113	20–200	5	35	41.66	^32^
Magnetized Tea waste& Methylene blue	5–30	0.025	RT	3.06	^39^
Polyaniline@oak acorn& Orange G	500	0.75	25	172.67	^35^
Modified oak pericarp& Rhodamine B	10–200	0.75	20	198.53	^55^
Modified oak pericarp& Rose Bengal	10–200	1	20	42.55	^55^
ACQC& BXGRL	27–336	1	25	222.22	Current work
ACQC& RV3R	25–291	1	25	106.38	Current work
WTSO& BXGRL	28–303	1	25	344.83	Current work
WTSO& RV3R	25–308	1	25	147.06	Current work

*RT: Room temperature, *m*: Adsorbent dosage.

The best uptake capacities calculated from Langmuir isotherm model were obtained for the removal of BXGRL toxic dye with two biosorbents examined in this work. WTSO exhibited the best uptake capacity for BXGRL with a very high value of 344.83 mg/g at only 25 °C. At higher temperatures it was found that the uptake capacities of the other biosorbent-dye sets studied in this work increased. The biosorbents in this study were used without any modification. There is no chemical or heat activation of biosorbents. So, the results showed that the uptake capacities of current biosorbents were very high when compared to the other biosorbents used with and without modifications in the literature. These high sorption capacities obtained with these biosorbents without any pretreatment have shown that they are highly efficient, economical and environmentally friendly and can be counted among the promising biosorbents for the future. Also, since the wastewater used in this study was prepared synthetically, the effect of other salts that may be present in the wastewater was neglected. Although the results obtained here show very high removal efficiencies and retention capacities, it should be considered that there may be some decrease in practical applications, and it is recommended that further studies should be carried out with column tests using real wastewater in the future to make these adsorbents as commercial materials. Furthermore, in a study that is conducted to remove toluidine blue and basic yellow 28 dyes, the ionic strength was assessed using NaCl solution and it was found that the removal efficiencies decreased negligibly^56^.

### Pt-Co color tests for comparing with legislations

The discharge of colored wastewaters into the aquatic environment is subject to legislation in Türkiye, as in many countries around the world. According to the Water Pollution Control Regulation^57^ in Türkiye, the discharge limit value for colored wastewater from textile industries is 280 Pt-Co. The values of Pt-Co for the initial dye concentrations in this study and real textile wastewaters were much higher than 500 Pt-Co. In experimental studies, at pH 8, 0.1 g biosorbent dosage, and average dye concentration of 49 mg/L, where good efficiency and uptake capacities were obtained, 96% efficiency was obtained with ACQC for BXGRL dye, while 98% removal efficiency was achieved with WTSO. In RV3R dye, removal efficiency was found to be 89% with ACQC, while this efficiency reached 92% with WTSO. When the efficiencies are examined, it is thought that the wastewater was successfully treated, but the adequacy of treatment according to the discharge limits of the wastewater creates a question mark. In this context, the Pt-Co color tests performed to answer this situation, clearly. As a result, the Pt-Co values with ACQC and WTSO for BXGRL dye were determined as 123 and 68, respectively. In RV3R dye, the Pt-Co values exhibited by ACQC and WTSO biosorbents after treatment were found to be 307 and 229, respectively. According to these results, the value of 280 was achieved in all sets except the RV3R-ACQC. It can be said that even in the RV3R-ACQC set, the value of 280 was not exceeded much. Thus, the effectiveness of biosorbents in terms of discharge limits has been demonstrated.

## Conclusions

Biosorption studies were conducted using two innovative biosorbents that had not been previously tested alone or in combination with the dyes used in this study, according to the literature. This study focused on biosorbent characterization and biosorption mechanisms, determination of optimal operating parameters, comparison of the biosorbents with those in the literature, and evaluation of whether wastewater discharge criteria were met. The removal of toxic dyes frequently used in the textile industry with the selected biosorbents was investigated in detail. As a result of the studies, it was concluded that both ACQC and WTSO biosorbents played a highly effective role in the removal of toxic BXGRL and RV3R dyes. Characterization experiments revealed the surface structures of the biosorbents and various functional groups such as carboxylate, hydroxyl, carbonyl etc. that could play an active role in treatment. Kinetic experiments indicated that the biosorption process was based on chemisorption and that diffusion alone could not be the rate-limiting step. Additionally, it was observed that biosorption occurred as a monolayer in all sets except for the RV3R biosorption with WTSO. The analyzed sorption capacities in this work were found to be quite high for raw biosorbents. The biosorbents demonstrated increased removal capacities at higher concentrations with rising temperatures, while the effect of temperature was negligible at low initial dye concentrations. The Pt-Co tests, typically used for discharge permits in real wastewater discharges, applied to the treated wastewater in this study showed that the color quality of the effluent was suitable for discharge. In this context, it was demonstrated that both biosorbents used in the study were highly effective in dye removal and showed promise as environmentally friendly alternative materials for sustainable waste management.

## Data Availability

Data will be made available on request.
